# Weight Loss in Patients Waiting for Total Hip Arthroplasty: Fiber-Enriched High Carbohydrate Diet Improves Hip Function and Decreases Pain before Surgery

**DOI:** 10.3390/jcm10184203

**Published:** 2021-09-17

**Authors:** Francesca Cannata, Alice Laudisio, Fabrizio Russo, Luca Ambrosio, Gianluca Vadalà, Marco Edoardo Cardinale, Chiara Bartolomei, Gabriella Iannone, Nicola Napoli, Rocco Papalia

**Affiliations:** 1 Department of Endocrinology and Diabetes, Campus Bio-Medico University of Rome, Via Álvaro del Portillo 200, 00128 Rome, Italy; f.cannata@unicampus.it (F.C.); bartolomeichiara@hotmail.it (C.B.); gabriella.iannone17@libero.it (G.I.); n.napoli@unicampus.it (N.N.); 2 Unit of Geriatrics, Department of Medicine, Campus Bio-Medico University of Rome, Via Álvaro del Portillo 200, 00128 Rome, Italy; 3 Department of Orthopaedics and Trauma Surgery, Campus Bio-Medico University of Rome, Via Álvaro del Portillo 200, 00128 Rome, Italy; fabrizio.russo@unicampus.it (F.R.); l.ambrosio@unicampus.it (L.A.); g.vadala@unicampus.it (G.V.); marco.cardinale.96@gmail.com (M.E.C.); r.papalia@unicampus.it (R.P.)

**Keywords:** fiber, diet, total hip arthroplasty, total hip replacement, obesity, elderly

## Abstract

The impact of obesity on clinical outcomes following joint replacement procedures is resounding. Therefore, multiple strategies to achieve a substantial weight loss before surgery are needed in obese patients. The aim of the study was to test the effect of a fiber-enriched high carbohydrate (FEHC) diet on the reduction in body weight and pain in elderly obese patients undergoing total hip arthroplasty (THA). Sixty-one candidates for THA were included in our study. Prior to the procedure, the participants have been randomly assigned to a 3-month diet intervention (FEHC diet or free diet). Anthropometric measures and food questionnaires were collected at the enrollment and after 3 months. The Oxford Hip Score (OHS), the Hip disability and Osteoarthritis Outcome Score (HOOS) and the Western Ontario McMaster Universities OA Index (WOMAC) were administered at baseline and before surgery. A statistically significant variation of weight was found in the FEHC diet group (−3.7 kg, −4.4–−2.5) compared to the control group (−0.2 kg; −1.4–1.7; *p* < 0.0001), as well as significant improvements in the OHS (*p* < 0.0001), the HOOS (*p* < 0.0001) and the WOMAC (*p* < 0.0001) questionnaires. According to the results of the study, the FEHC diet in obese patients undergoing THA might help weight loss and improve related anthropometric parameters as well as hip function and pain.

## 1. Introduction

Obesity is one of the preventable causes of death and accounts for over 2.5 million deaths annually worldwide [1]. The prevalence of obesity is expected to progressively rise with an increasing trend in the younger population. It is expected to determine an inevitable increase in hip osteoarthritis (OA) and an exponential increase in total hip arthroplasty (THA) procedures [2]. Indeed, the higher mechanical load in weight-bearing joints negatively impact on joint homeostasis [3]. Metabolic disorders and potential systemic mediators related to obesity might also contribute to joint degeneration and favor the development of OA [4,5].

THA is now the third most commonly performed operation in Western countries [6]. It involves the surgical remodeling of the hip to restore congruity, relieve pain and improve joint function. The demand for THA has increased over the years due to the proven success of these procedures to ameliorate the quality of life of patients [7]. Several conditions and risk factors are responsible for this increase, among which the most important are mechanical factors, including higher body mass index (BMI), traumatic injuries, malformations, heavy physical stress at work and aging [6].

Patients with a higher BMI have an increased risk of peri-operative complications, prolonged duration of surgery, extended rehabilitation, and convalescence, as well as various short-term complications, especially infections and dislocations [8,9,10,11,12], and a shortened duration of the prosthesis. Moreover, in terms of efficacy, overweight and obese patients seem to benefit from arthroplasty as much as non-obese patients, despite a slower recovery and lower function-related scores [13,14,15,16,17].

Therefore, weight loss in patients undergoing THA is desirable not only before surgery, but especially in the postoperative period. However, weight loss and lifestyle change are an insurmountable obstacle in people with obesity and diabetes [18]. Several studies have underlined the beneficial effects of preoperative dietary interventions on weight loss, the reduced risk of post-operative infections and improved glycemic control before total joint arthroplasty [19,20,21]. Over time, several dietary approaches for weight loss in people with obesity have been proposed, each one characterized by different contents of macronutrients. Several studies have confirmed the central role of a high-fiber diet in weight loss. Undoubtedly, the most important and recognized is the Mediterranean diet, characterized by high consumption of fruit, vegetables, nuts, cereals and olive oil, as well as a moderate consumption of fish and poultry and a low consumption of sweets, red meat and dairy products [22].

However, very few studies investigated the role of specific diet administration in the preoperative stage on reducing body weight and pain in elderly obese patients undergoing THA. Based on the proven beneficial effect of a high daily consumption of dietary fiber, the aim of this study was to assess the efficacy of a fiber-enriched high carbohydrate (FEHC) diet on reducing body weight, pain and hip function in elderly obese patients undergoing THA.

## 2. Materials and Methods

### 2.1. Patients

A cross sectional study was conducted at the Department of Orthopaedic and Trauma Surgery of the Campus Bio-Medico University Hospital, Rome, Italy. Patients aged between 65–85 years old, with a BMI > 30 kg/m^2^ and affected by primary hip OA and scheduled for THA were included in the study between 2019 and 2020. Patients suffering from secondary hip OA, femur neck fracture and osteonecrosis of the femoral head were excluded. The study protocol has been approved by the Ethical Committee of Campus Bio-Medico University of Rome.

**Figure 1:**
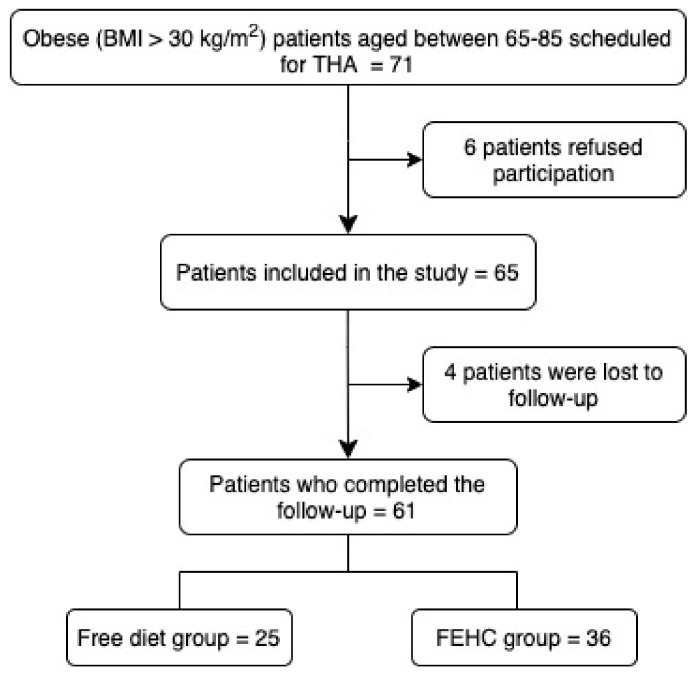
Patient inclusion flowchart.

All patients were allocated in the following two groups using computer-generated random numbers (Microsoft Excel v. 16.48, Microsoft, Redmond, WA, USA) at the time of inclusion: (1) a free diet group and (2) an FEHC diet group. A total of 61 patients scheduled for THA were enrolled in the study, 36 in an FEHC group and 25 in the free diet group (Figure 1). Patients in the free diet group received general information about a healthy diet but not a specific diet, whereas individuals included in the FEHC diet group followed a specific diet for 3 months before surgery with controlled micronutrients and a restricted caloric content of 1700 kcal. The macronutrient composition was 72% from carbohydrates, 10% from vegetable proteins and 18% from fats. Complex carbohydrates (derived from whole grains, legumes, fruit, and vegetables) were 60% (255 g). Simple sugars (integrated with seasonal fresh fruit) were 12% of total carbohydrates (54 g). Daily fiber content was 40 g per 1000 kcal; animal products and or added sugars were not included.

### 2.2. Study Variables and Outcome Measurements

Age, gender, height, weight, and cholesterol level in a blood sample were annotated at the baseline on enrolment and at the follow-up visit after 3 months. BMI was calculated as weight (kg) divided by height squared (m^2^). Adherence to diet was investigated through a periodic monthly follow-up visit and a weekly telephone call.

Functional outcomes were assessed at the baseline after 3 months of dietary intervention with the Oxford Hip Score (OHS), the Hip disabilities and Osteoarthritis Outcome Score (HOOS) and the Western Ontario and McMaster Universities (WOMAC) Osteoarthritis Index. OHS is a joint-specific patient-reported outcome measure tool to evaluate disability in patients undergoing THA (score range = 12–60) [23]. HOOS is a 40-item questionnaire to assess patient-relevant outcomes in five separate subscales (pain, symptoms, activity of daily living, sport and recreation function and hip related quality of life; score range = 0–100) [24]. The WOMAC Osteoarthritis Index is a self-administered questionnaire consisting of 24 items divided into the following 3 subscales: pain, stiffness, and physical function (score range = 0–96) [8]. In addition, the minimal clinically important difference (MCID) of the adopted scales was calculated using a distribution-based method [25].

### 2.3. Statistical Analysis

Statistical analyses were performed using Statistical Package for Social Science (SPSS) for Mac 26.0. Differences were considered significant at the *p* < 0.05 level. Data of continuous variables are presented as mean values ± standard deviation (SD). Median values with inter-quartile ranges were provided for non-normally distributed variables. Analysis of variance (ANOVA) for normally distributed variables was performed according to diet; otherwise, the nonparametric Mann–Whitney U H test was adopted. The two-tailed Fisher exact test was used for dichotomous variables. Eventually, body parameters and questionnaire scores were compared before and after the dietetic intervention by using the Wilcoxon signed-rank test.

## 3. Results

The mean age of the study population was 73 ± 6 years, and 36 (59%) were female. Additionally, 36 subjects were randomized to the FEHC diet, while 25 participants received the control free diet. The main characteristics of the population are shown in Table 1; specifically, the patients who followed the FEHC diet had significantly higher scores in the HOOS questionnaire at both baseline and follow-up times, and lower scores in the WOMAC questionnaire at follow-up as compared with the controls. The variations in body parameters and questionnaire scores from baseline to follow-up according to the FEHC diet are depicted in Table 2, while the improvement in body parameters and questionnaire scores according to the FEHC diet are described in Table 3.

**Figure 2:**
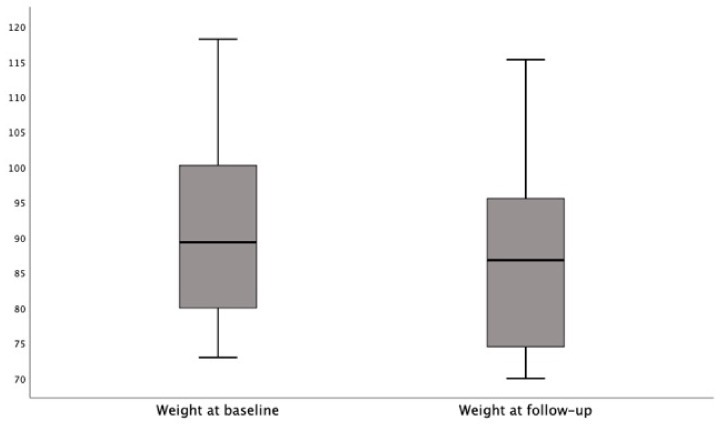
The follow-up weight medians were significantly lower than the baseline medians in participants on FEHC diet.

The Wilcoxon signed-rank test indicated that the follow-up weight medians were significantly lower than the baseline medians in the participants on the FEHC diet (Z = −5.1; *p* < 0.0001. Figure 2); conversely, the weight medians did not differ from baseline to follow-up in the participants on the free diet (Z = −0.3; *p* = 777).

The Wilcoxon signed-rank test indicated that the follow-up BMI medians were significantly lower than the baseline medians in the participants on the FEHC diet (Z = −5.2; *p* < 0.0001); conversely, the weight medians did not differ from baseline to follow-up in the participants on the free diet (Z = 0.6; *p* = 954). The Wilcoxon signed-rank test indicated that the follow-up OHS scores (median 38) were significantly higher than the baseline scores (median: 31) in the participants on the FEHC diet (Z = 4.2; *p* <0.0001); conversely, the follow-up OHS scores (median: 34) were significantly lower than the baseline scores (median: 37) in the participants on the free diet (Z = −3.0; *p* = 0.002).

The Wilcoxon signed-rank test indicated that the follow-up HOOS scores were significantly higher than the baseline scores in the participants on the FEHC diet (Z = 2.1; *p* = 0.033); conversely, the follow-up HOOS scores were significantly lower than the baseline scores in the participants on the free diet (Z = −3.6; *p* < 0.0001).

**Figure 3:**
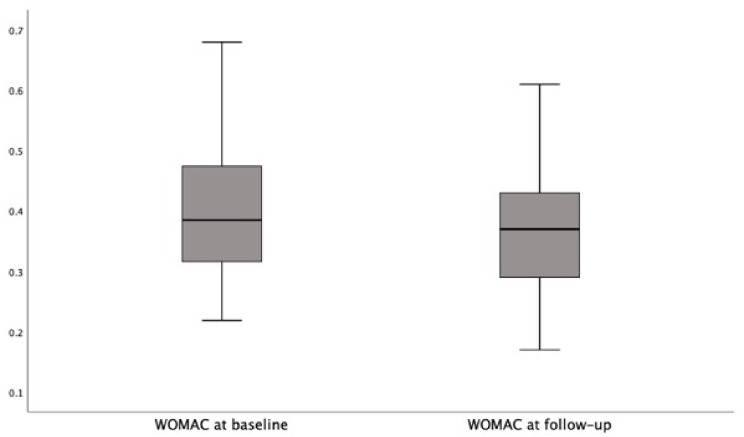
The follow-up WOMAC scores in participants on FEHC diet.

The Wilcoxon signed-rank test indicated that the follow-up WOMAC scores (median: 0.37) were significantly lower than the baseline scores (median: 0.38) in the participants on the FEHC diet (Z = −5.1; *p* < 0.0001. Figure 3); conversely, the follow-up WOMAC scores (median 0.43) were significantly higher than the baseline scores (median: 0.39) in the participants on the free diet (Z = 4.2; *p* < 0.0001).

Additionally, MCID calculation documented a small effect (Table 4).

## 4. Discussion

In this study, we found a close correlation (*p* < 0.001) between the use of the FEHC diet and the improvement of anthropometric parameters. The FEHC diet, in three months of administration, produced a significant reduction in body weight and BMI compared to the free diet group. The FEHC diet led to an improvement in OHS (*p* < 0.0001), HOOS (*p* < 0.0001) and WOMAC (*p* < 0.0001) scores even before undergoing THA, suggesting the efficacy of weight loss in the optimization of hip symptoms and motility. Nevertheless, dietary intervention in our population had only small effects considering the MCID results. However, it is possible that the short follow up might contribute to these data.

Obesity and OA are two interconnected healthcare problems affecting a large proportion of the adult population worldwide. The increasing weight of the population will lead to nearly 1.3 billion and 573 million adults being overweight and obese by 2030, respectively. Moreover, OA increases as the population ages, representing a leading cause of chronic pain and disability among older people [26].

Considering the high impact of obesity on the complication rate and outcomes, weight loss is warmly encouraged before THA, as a decrease in body weight > 10% is associated with improved pain and functional scores [27]. In some cases where rapid and consistent weight loss is desirable, bariatric surgery has been employed to optimize morbidly obese patients before THA. However, a meta-analysis from Smith et al. [28] reported that preoperative bariatric surgery did not improve the risk of infection, deep venous thrombosis, pulmonary embolism, and revision surgery. Therefore, the role of bariatric surgery for managing obesity before THA surgery remains controversial.

Obesity leads to low-grade systemic inflammation, and weight reduction can reduce adipose tissue and restore normal secretion patterns [29,30].

The reduction in pain may be associated with a reduction in weight (an important risk factor for OA in the lower joints for increasing the biomechanical stress) and a modification of the inflammation parameters that seem to increase hip OA [2].

Several studies have found a clear relationship between weight loss in obese adults and pain reduction. In the guidelines from the American College of Rheumatology [31] and European League Against Rheumatism [32], weight loss and physical exercise are recommended in overweight or obese patients with OA.

Lui et al. investigated the effect of non-surgical, non-pharmacological weight loss interventions in patients who are obese prior to THA. The authors concluded that there is insufficient evidence to support the recommendation that patients who are obese lose weight (≥5%) within the year prior to THA [19].

In a systematic review, Seward et al. investigated preoperative nonsurgical weight loss interventions before THA and knee arthroplasty. The conclusion of this study is that the available evidence indicates that short-term, nonsurgical, preoperative weight loss interventions before THA can produce statistically significant weight loss and a decreased BMI that may help some patients with severe obesity achieve a BMI below the standard 40 kg/m^2^ cut-off for surgery. However, it remains unknown if the amount of weight loss from these interventions is clinically significant and sufficient to improve outcomes after THA [20].

The role of fiber in the regulation of body weight has already been discussed in the literature. Dietary fiber is defined as an enzymatically undegradable and non-absorbable component of the diet. Fibers can be mainly divided into the two different types: soluble and insoluble. The difference is essentially in the capacity of the fiber itself to dissolve in water; indeed, most vegetables contain both types with different proportions [33].

Experimental studies have shown how a diet rich in fiber reduces the absorption of free fatty acids by using them as energy in the fermentation process of the fibers in the intestine. In addition, dietary fibers have the ability to retain water; this leads to a lowering of the energy/weight ratio of the ingested food, stimulating the sense of satiety despite a reduced energy intake and, thus, reducing voluntary food intake [34,35].

Many physiological mechanisms of the action of dietary fiber in weight loss have been studied. Fiber appears to reduce food intake through the increased effort and time it takes to chew it [36]. In a study by Birketvedt et al., the addition of dietary fiber in a low-calorie diet significantly improved weight loss, with the placebo group presenting a 5.8 kg weight reduction compared to an 8.0 kg weight reduction in the group with fiber supplementation [37]. Studies of postmenopausal women have also shown that the introduction of a higher fiber content within a very low-fat diet increases weight loss [38]. In a study by Te Morenga et al., a diet rich in fiber was demonstrated to reduce hunger and increase patient compliance compared to a protein-rich diet. Moreover, besides having a statistically significant effect on body weight or comparable to the latter, it has shown an important association with a reduction in blood pressure [39]. A key aspect of a higher fiber diet is also linked to a reduction in cholesterol levels. In a study published in the Journal Of The American College Of Nutrition [40], a diet rich in fiber was shown to reduce low-density lipoprotein (LDL) cholesterol levels not only in dyslipidemic patients, but also in normolipidemic subjects. These aspects have also been correlated with the risk of coronary events: in a study by Anderson et al., a clear inversely proportional relationship was demonstrated between the amount of soluble fiber introduced with the diet and the incidence of coronary events [41].

This study has some limitations. The first limitation was related to randomization, and this produced a selection bias in the enrollment of the patients that was clear in the significant difference in the HOOS score. Additionally, no data regarding the sub-score of the adopted scales were recorded; thus, only the total final scores were shown. Ultimately, we cannot determine the adherence to the diet; however, this is issue is common in most studies on diet. Even though the baseline and follow-up weights indicated that the high-fiber diet does not produce any significant weight loss as compared to the free diet, the intra-group variation was significant; this might reflect an insufficient sample size. Thus, further, larger studies are needed to support our hypotheses.

## 5. Conclusions

The present study has shown that the administration of an FEHC diet may promote an improvement of body weight and BMI in obese elderly patients with hip OA scheduled for THA. Indeed, the patients who followed an FEHC diet for three months lost weight and improved their clinical scores before surgery. Indeed, losing weight is crucial in order to avoid surgical complications as well as boost the postoperative recovery. Therefore, a dietary approach based on an FEHC diet for obese elderly patients may be successful to lose weight and improve clinical outcomes in this specific subset of patients. Although, after three months of the diet, the follow up BMI and weight did not differ significantly in the two groups; there was an improvement in cholesterol and on the WOMAC questionnaire that investigates symptoms such as pain, stiffness, and functional autonomy.

## Figures and Tables

**Table 1:** Characteristics of the study population according to FEHC diet.

	FEHC Diet (*n* = 36) Mean (SD) or Median (IQR) or *n* (%)	Free Diet (*n* = 25) Mean (SD) or Median (IQR) or *n* (%)	*p*
Age (years)	74 (7)	72 (6)	0.146
Gender (female)	23 (64)	13 (52)	0.431
Weight at baseline (kg)	89.3 (80.0–100.2)	91.5(84.0–95.6)	0.889
Weight at follow-up (kg)	86.8 (74.5–95.5)	89.0 (82.6–96.2)	0.205
BMI at baseline (kg/m^2^)	33.4 (31.8–37.5)	32.3 (30.9–34.3)	0.070
BMI at follow-up (kg/m^2^)	31.7 (30.1–35.1)	32.0 (30.8–33.8)	0.832
Total cholesterol at baseline (mg/dL)	198 (40)	202 (31)	0.721
Total cholesterol at follow-up (mg/dL)	180 (28)	203 (34)	0.006
OHS questionnaire baseline	31 (27–44)	37 (34–46)	0.108
OHS questionnaire follow-up	38 (30–46)	34 (29–41)	0.199
HOOS questionnaire baseline	65 (53–88)	32 (24–39)	<0.0001
HOOS questionnaire follow-up	65 (54–91)	29 (23–31)	<0.0001
WOMAC questionnaire baseline	0.38 (0.32–0.47)	0.39 (0.33–0.47)	0.826
WOMAC questionnaire follow-up	0.37 (0.29–0.43)	0.43 (0.37–0.49)	0.005

**Table 2:** Variation in anthropometric parameters and questionnaire scores from baseline (T0) to follow-up (T1) according to FEHC diet.

	FEHC Diet (*n* = 36)	Free Diet (*n* = 25)	*p*
Weight (kg)	−3.7 (−4.4–−2.5)	−0.2 (−1.4–1.7)	<0.0001
BMI (kg/m^2^)	−1.5 (−2.0–−0.8)	0.1 (−0.7–1.2)	<0.0001
Total cholesterol (mg/dL)	18 (7–33)	−9 (−12–−2)	<0.0001
OHS questionnaire	3 (2–7)	−4 (−10–0)	<0.0001
HOOS questionnaire	1 (−1–2)	−3 (−4–−2)	<0.0001
WOMAC questionnaire	−0.05 (−0.05–−0.02)	0.03 (0.02–0.05)	<0.0001

**Table 3:** Improvement in body parameters and questionnaire scores according to FEHC diet.

	FEHC Diet (*n* = 36) *n* (%)	Free Diet (*n* = 25) *n* (%)	*p*
Weight loss	35 (97)	14 (56)	<0.0001
BMI loss	35 (97)	14 (56)	<0.0001
Reduced total cholesterol	32 (89)	5 (20)	<0.0001
OHS questionnaire	31 (86)	6 (24)	<0.0001
HOOS questionnaire	24 (67)	3 (12)	<0.0001
WOMAC questionnaire	18 (50)	1 (4)	<0.0001

**Table 4:** Range of scores and minimal clinically important differences (MCIDs) of adopted scales.

	Range of Score	MCID
OHS questionnaire	12–60	0.12
HOOS questionnaire	0–100	0.45
WOMAC questionnaire	0–96	0.10

## Data Availability

The data presented in this study are available on request from the corresponding author.
